# Scientific names of organisms: attribution, rights, and licensing

**DOI:** 10.1186/1756-0500-7-79

**Published:** 2014-02-04

**Authors:** David J Patterson, Willi Egloff, Donat Agosti, David Eades, Nico Franz, Gregor Hagedorn, Jonathan A Rees, David P Remsen

**Affiliations:** 1 School of Life Sciences, Arizona State University, Tempe, Arizona 85287, USA; 2 Plazi, Zinggstrasse 16, 3007 Berne, Switzerland; 3 Museum für Naturkunde, Leibniz-Institut für Evolutions- und Biodiversitätsforschung, 10115 Berlin, Germany; 4 Illinois Natural History Survey, University of Illinois, Champaign, Illinois 61820, USA; 5 National Evolutionary Synthesis Center (NESCent), Durham, North Carolina 27705, USA; 6 Marine Biological laboratory, Woods Hole, MA 02543, USA

**Keywords:** Scientific names, Taxonomy, Copyright, Intellectual property rights, Name-based infrastructure, Big data

## Abstract

**Background:**

As biological disciplines extend into the ‘big data’ world, they will need a names-based infrastructure to index and interconnect distributed data. The infrastructure must have access to all names of all organisms if it is to manage all information. Those who compile lists of species hold different views as to the intellectual property rights that apply to the lists. This creates uncertainty that impedes the development of a much-needed infrastructure for sharing biological data in the digital world.

**Findings:**

The laws in the United States of America and European Union are consistent with the position that scientific names of organisms and their compilation in checklists, classifications or taxonomic revisions are not subject to copyright. Compilations of names, such as classifications or checklists, are not creative in the sense of copyright law. Many content providers desire credit for their efforts.

**Conclusions:**

A ‘blue list’ identifies elements of checklists, classifications and monographs to which intellectual property rights do not apply. To promote sharing, authors of taxonomic content, compilers, intermediaries, and aggregators should receive citable recognition for their contributions, with the greatest recognition being given to the originating authors. Mechanisms for achieving this are discussed.

## Findings

### Introduction - names for biodiversity informatics

Scientific names of organisms identify units of biodiversity and have value in biodiversity informatics [1]. Their almost universal use for over 250 years allows them to be used as metadata to index and organize biodiversity information. Their use in a names-based infrastructure will help the transformation of Biology into a ‘big data’ discipline [2]. To fulfill this role, the infrastructure needs to have access to all names that have ever been used for all taxa. It must include variant and erroneous spellings of the same names, all synonyms, common names, and surrogates for names (such as molecular barcodes) if it is to link all content on the same species irrespective of what name or spelling was used in the source. Names are often organized in hierarchical classifications that serve as tallies of how much biodiversity has been described. Hierarchies have value because they can be used to browse or navigate content, and to launch aggregative searches that transform a query about parrots into a query for every known parrot using every known name.

Lists of names and species are compiled by taxonomists in compliance with the rules of Codes of Nomenclature - consensual frameworks without legal standing [3-6]. Various licensing conditions impede re-use of content [7], and create uncertainty as to how intellectual property rights apply to names and lists of names. This issue was the subject of a workshop held in Tempe, Arizona (April, 2013) that brought together biologists with interests in names and legal authorities with expertise in intellectual property rights. Additional input was sought from the ‘Taxacom’ Biological Systematics Discussion List [8].

A vision is that the ‘Big New Biology’ will provide systematists and those who depend on their work, such as ecologists and phylogeneticists, with free and easy access to names and taxonomic content. That will make taxonomy more relevant. Yet, at this time, we do not have a single complete list of all species described to date nor do we have a single point of access to all taxonomic information [9]. Taxonomic information is overseen by a community of taxonomists estimated as being between 6,000 and 50,000 strong [10-12]. Most new taxonomic information first appears in scientific publications, although web-sites and on-line registration of new names [13,14] are changing this pattern. The first step in the process of building a list of names begins by cataloguing the nomenclatural acts that created new names. Nomenclators and registries, such as ZooBank, Index Fungorum, the International Plant Name Index, and Index Nominum Genericorum, compile information on these code-compliant actions (where ‘code’ refers to codes of nomenclature). Taxonomists periodically review taxonomic and nomenclatural knowledge in their area of expertise. Despite the increasing availability of literature on-line through initiatives such as the Biodiversity Heritage Library (BHL), the task of reviewing the legacy literature for uncompiled or overlooked nomenclatural and taxonomic acts is huge. Some of this information is made available through taxonomic web sites and is drawn together by aggregators such as Catalogue of Life, World Registry of Marine Species, or the Interim Register of Marine and Nonmarine Genera. The Catalogue of Life is one of the most visible compilations, and claims about two thirds of known extant species. It excludes extinct species excepting recently extinguished ones. It offers a single perspective in each taxonomic domain, excluding all alternative perspectives of which there are many. Currently, those who require comprehensive taxonomic knowledge have no single place to obtain it but must explore the taxonomic literature and visit numerous on-line specialist web-sites.

The relevant content for a names-based infrastructure is in nomenclators, registries, the scientific literature, checklists, classifications, taxonomic revisions and monographs, biodiversity databases and web sites, and in the compilations of aggregators. All lists include material from multiple sources. Each source uses names in a context. For example, nomenclators and registries indicate the correct orthography of each scientific name, accompany it with the name of its author, the date when the name was introduced, and a citation pointing to where it was first used. This may be in the form of a condensed micro-citation. Nomenclator Zoologicus [15] has the entry for the crab genus *Cancer*: “Cancer Linnaeus 1758 Syst. Nat., ed. 10, 625; 1767, ed. 12, 1038”. The taxonomic context may be included (as in the example above, *Cancer* is annotated with the term ‘crust’ indicating that it is a member of the Crustacea). Nomenclators develop lists of scientific names of taxa, but are not lists of taxa because a nomenclator makes no evaluation as to the taxonomic status of a name.

Checklists are listings of names of species relevant to a particular context - such as species of birds found in a particular geographical location, or the ‘red list’ of endangered species [16]. Such lists may not be taxonomically authoritative and may or may not include author and date information. Annotated checklists may cite taxonomic treatments or address the identification of specimens, synonymy and how to distinguish the taxon.

Taxonomic treatments are reviews that identify the taxa that are accepted by the authors of the treatment. The names of species that are accepted are usually placed in taxonomic hierarchies, may be accompanied with synonymy statements, may have pointers to usages and chresonyms [17], may include original taxonomic opinions not published elsewhere, may include descriptions, may have additional data such as molecular data, images, distribution maps, and extensive citation lists. The layout of such treatments has been trending to a standard ‘revisionary’ or ‘monographic’ style that uses a taxonomic hierarchy, presents names, authors, nomenclatural acts, synonymy statements, materials observed, descriptions, comments, and references [18,19].

It is from the collective efforts of nomenclaturalists and taxonomists that a names-based infrastructure must gain the names it relies upon. Taxonomists will make more progress by making incremental additions to a common knowledge resource rather than duplicating the works of others. Yet co-operation is slowed by restrictions on the re-use of existing knowledge that are imposed through licenses (see Table 1). There is a diversity of licensing arrangements that creates uncertainty among users as to the legality of re-using names from other sources. Uncertainties delay progress because potential users avoid actions that might cause offence or undermine future collaborations.

**Table 1:** Examples of statements about re-use of names, nomenclatural, and/or taxonomic content

•	**Algaebase,** taxonomic information about algae [20]. “The images, information and data on this site are not in the public domain and are the property of the copyright owners. The data may not be downloaded or replicated by any means, manually or mechanically, including copying and pasting into theses, papers and other publications, and extraction by any means, manually or electronically. Any copying of the data or images, be it commercial or non-commercial (including non-profit), educational or non-educational, without the written permission of the copyright owner and payment, if requested, may result in legal action, including legal action involving the service provider or publisher.
•	The **BioSystematic Database of World Diptera**[21]. “The BDWD operates under US Law, especially the fair-use provisions of the Copyright Law. As the major supporter of the project is an US Government agency, the BDWD is without copyright”.
•	**Catalogue of Life,** a taxonomic compilation [22]. “This online database is copyrighted by Species 2000 on behalf of the Catalogue of Life partners. Use of the content (such as the classification, synonymic species checklist, and scientific names) for publications and databases by individuals and organizations for not-for-profit usage is encouraged, on condition that full and precise credit is given at three levels on all occasions that records are shown”.
•	**Index Fungorum,** Nomenclator of fungal names, [23]. “The custodians, either collectively or individually, claim no IPR over the compilation, which correctly reside with the many contributors, including the custodians”.
•	**IPNI,** A botanical nomenclator [24]. “This database and its contents are © copyright the Plant Names Project. All rights reserved”. but also “Copies, including electronic, may be made of the data held within this database for your own use or for use within your organisation”.
•	The **Plant List**[25]. “This online database is copyrighted by the Royal Botanic Gardens, Kew and Missouri Botanical Garden on behalf of The Plant List (TPL) and its contributors. The Plant List by Missouri Botanical Gardens and the Royal Botanic Gardens, Kew, on behalf of The Plant List and its contributors is licensed under a Creative Commons Attribution-NonCommercial-NoDerivs 3.0 Unported License. Use of the content (such as the classification, synonymised species checklist, and scientific names) for publications and databases by individuals and organizations for not-for-profit usage is encouraged, on condition that full and precise credit is given to The Plant List and the conditions of the Creative Commons Licence are observed”. The no-derivative component of the license has been criticized by Page [26] and Heim [27] because “An ND license renders a dataset useless”. At the time of writing, the licensing of the Plant List is being changed.
•	**Thomson Reuters Index of Organism Names**[28]. “All content provided on this site is owned by or licensed to Thomson Reuters and/or its affiliates (the “Thomson Reuters Content”) and protected by United States and international copyright laws. Thomson Reuters and its licensors retain all proprietary rights to the Thomson Reuters Content. The Thomson Reuters Content may not be reproduced, transmitted or distributed without the prior written consent of Thomson Reuters”.
•	**WORMS World Registry of Marine Species**[29]. “Unless otherwise stated, these web pages and associated information are free to use on condition that they are cited (CC-BY). A recommended citation style is provided on each page. We do not permit the re-distribution of the entire database unless by prior written agreement”.

### The legal context

Data providers who seek to impose conditions on the use of data may refer to intellectual property rights such as copyright, database rights, or contract laws as the basis of restrictions.

Intellectual property rights are established by the 1967 Convention Establishing the World Intellectual Property Organization (WIPO) [30]. WIPO deals with copyright, neighbouring rights (relating to performances, phonograms and broadcasts), patent rights that relate to inventions, scientific discoveries, industrial designs and trademarks. Of these, only copyright law is relevant to names and compilations of names. In Europe, Database Rights are also relevant.

International copyright law is based on the Berne Convention for the Protection of Literary and Artistic works [31]. It applies to ‘works’ but leaves each signatory state to determine its own definition of a work as long as it respects the Convention’s framework. Copyright in the US is regulated by the US Copyright Act of 1976 and the articles of Title 17 of the United States Code [32,33] and various emendations [34]. Section (§) 102 of this act declares that “copyright protection subsists (…) in original works of authorship fixed in any tangible medium of expression” but that “in no case does copyright protection for an original work of authorship extend to any idea, procedure, process, system, method of operation, concept, or discovery, regardless of the form in which it is described, illustrated or embodied in such work”. Most copyright laws in European countries refer to a work, in different wordings, as an intellectual creation made by a human that is somehow original, new, or different when compared to pre-existing creations. The European Court of Justice has ruled that a product, in order to claim copyright protection, has “to be original in the sense that it is its author’s own intellectual creation” [35]. In sum, the common understanding of a protected work is of an intellectual product that contains some minimal degree of authorship that makes the creation original and expresses free and creative choices in the production of the work. This originality refers to the form of presentation, not to the content. Copyright gives near-monopolistic control for (in most countries) the life-span of the creator plus 70 years.

Special rules may apply to databases. U.S.A. and European law differ in this regard. In 1996, the E. U. introduced database rights to provide legal protection of databases with Directive 96/9/EC [36]. The United Kingdom has “The Copyright and Rights in Databases Regulations 1997’ [37]. The E.U. protection is not part of copyright but is a sui generis (special case) right that applies whether copyright relating to the database exists or not. Databases may independently be subject to copyright restrictions based on creative elements. Database rights apply only to databases which show “that there has been qualitatively and/or quantitatively a substantial investment in either the obtaining, verification or presentation of the contents” (art. 7, Directive 96/9/EC). It allows the person who made the database, or the employer if the database was made by employees as part of their work, to prevent re-utilization of the whole or of a substantial part of the contents of the database. An attempt to introduce a similar right as an international convention was defeated at the WIPO Diplomatic Conference in 1996 [38,39] However, article 5 of the WIPO Copyright Treaty [40] states: ‘Compilations of data or other material, in any form, which by reason of the selection or arrangement of their contents constitute intellectual creations, are protected as such. This protection does not extend to the data or the material itself.’ Sui generis database rights do not exist in the U.S [41]. Database rights would be violated by unauthorized use of the whole or substantial part of the database, although exceptions may apply to use for research (the UK law states that the database right is not infringed when content is extracted for the purpose of illustration for teaching or research and not for any commercial purpose). The legal situation with non-creative elements of databases is not clear, as is evidenced by the Case Law example 3, below.

Even when copyright is applicable, copyright laws have clauses that allow for certain use of copyrighted material. The best known example is the “Fair-Use-clause” in the U.S. copyright act (Section 107) which states “the fair use of a copyrighted work, including such use by reproduction in copies or phonorecords or by any other means specified by that section, for purposes such as criticism, comment, news reporting, teaching (including multiple copies for classroom use), scholarship, or research, is not an infringement of copyright.” Fair use requires a case by case evaluation that often comes down to the argument of whether the fair use achieves the intent of copyright law to promote the “Progress of Science and useful Arts” [42]. In Europe, Article 5.3 of European Directive 2001/29/EC on the harmonisation of certain aspects of copyright states that *“Member States may provide for exceptions or limitations .... in the following cases: (a) use for the sole purpose of illustration for teaching or scientific research, as long as the source, including the author’s name, is indicated, .....”* and *“(n) use by communication or making available, for the purpose of research or private study, to individual members of the public by dedicated terminals”* at places such as publicly accessible libraries, educational establishments or museums which do not exist for economic or commercial advantage.

‘Sweat of brow’ is part of an argument that authors gain rights over their products because of an investment of effort, rather than because of a creative contribution. This position has a certain relevance to database protection in the EU, but has no merit in copyright. In a judgment of 1.3.2012, the European Court of Justice ruled explicitly that “sweat of brow” (in the terms of the Court “labour and skill”) cannot justify any copyright protection if the labour and skill do not express any originality in the selection or arrangement of data [43].

Data Use Agreements (DUA) provide a mechanism by which data providers may regulate the use of information independently of copyright limitations. Those agreements may involve a written contract, or may simply be said to have been agreed as a result of accessing content. Exactly what constitutes entering into a DUA is unclear and is not well tested in the courts. It is not clear if, for example, it is sufficient for the provider to place an appropriately worded “terms of use” statement on their web site for a DUA to be established. Such agreements are ‘relative rights’ through which two parties stipulate and agree on the terms of access and use of an object. Such agreements may be called “licenses”. If the agreement is not respected, the licensor can act against the licensee. A number of factors make this an uncertain area. Firstly, in the US, agreements between two parties supersede copyright rules, but this is not necessarily so in European law. Secondly, the licensor is unlikely to be the sole source of factual information making it hard for the licensor to prove abuse of the agreement. Finally, the legal status of agreements made as part of the process of gaining access to content through a web site or by statements on a web site is not clear. If a content provider wishes to limit the re-use of content, the most appropriate solution is to have an explicit and particular written agreement with each user.

### Case histories

There is no ‘simple bright line’ that distinguishes ‘right’ from ‘wrong’ in law. Rather, the law is open to debate, and its interpretation can be found as ‘case history’ in the judgments of courts of law. Disputed results lead to appeals, and cases may proceed to higher levels of courts where earlier results may be overturned or modified. Case history provides us with guidance as to how courts treat the law, can set precedents, or reveal differences of opinions among judges. A review of cases exposes arguments and allows uncertainties to be clarified. We are not aware of any case history that relates to scientific names of organisms. Three cases do provide context for names and compilations of names.

**Case 1. Feist Publications, Inc. v. Rural Telephone Service Co., 499 U.S. 340, 345–46, 111 S.Ct. 1282, 1287–88, 113 L.Ed.2d 358 (1991)**[44] refers to the re-use of facts (telephone numbers) by Feist publications from a compilation made by the Rural Telephone Service. The Rural Telephone Service objected to the re-use of information in their compilation. The original case found in favor of Rural but this was reversed on appeal by the Supreme Court. The Supreme Court stated that a compilation is copyrightable only if its content has been “selected, coordinated, or arranged *in such a way* that the resulting work as a whole constitutes an original work of authorship.” It made the point that there was nothing creative about arranging names alphabetically. Even if a compilation is copyrightable, it receives only limited protection, because the copyright does not extend to facts contained in the compilation (U.S.C 17 § 103(b)). This decision confirms that names as facts are not copyrightable and that copyright does not apply to a familiar arrangement of content.

**Case 2. American Dental Association, Plaintiff-Appellant, v. Delta Dental Plans Association, Defendant-Appellee. No. 96–4140. Argued May 30, 1997, decided September 30, 1997**[45] addresses whether a taxonomy (of dental procedures) is copyrightable. The original hearing concluded that no taxonomy was copyrightable. The conclusion was widely disputed and the appeal argued that things can be organized in different ways, with a pointer to biology. “So too with a taxonomy - of butterflies, legal citations, or dental procedures. Facts do not supply their own principles of organization. Classification is a creative endeavor. Butterflies may be grouped by their color, or the shape of their wings, or their feeding or breeding habits, or their habitats, or the attributes of their caterpillars, or the sequence of their DNA; each scheme of classification could be expressed in multiple ways.” The appeal court set aside the original judgment, but did not provide an alternative judgment. The point that ‘classification is a creative endeavor’ deserves comment. It would have been more appropriate to say that a classification MAY BE a creative endeavor. As noted in Rural v. Feist: “there is nothing remotely creative about arranging names alphabetically in a white pages directory”. Similarly, the US code precludes “any idea, procedure, process, system” from copyright cover. Biologists regard taxonomies (classifications) as systems because they adopt familiar hierarchically nested sets (Kingdom, Phylum, Class, Order, Family, Genus, Species, etc.), and because names and other elements are written out following standard conventions. Taxonomic classifications are not creative in the sense of the law and so cannot acquire copyright protection. This has not been tested in court.

**Case 3. Football Dataco Ltd. and Others vs. Yahoo! UK Ltd. and Others; European Court of Justice, C-604/10, 1.3.2012**[46] refers to fixture lists for the English and Scottish football leagues. Football Dataco and Others claimed that they own, in respect of the English and Scottish football league fixture lists, a ‘sui generis’ right pursuant to Article 7 of Directive 96/9, a copyright pursuant to Article 3 of that directive, and a copyright under United Kingdom intellectual property legislation. Yahoo and Others did not accept that such rights exist in law, arguing that they are entitled to use the lists in the conduct of their business. The initial judge found that the preparation of football fixture lists is not purely mechanistic unlike, for instance, the compilation of a telephone directory. Rather it requires judgment and skill to balance many competing requirements. The judge held that the lists are eligible for copyright protection under Article 3 of Directive 96/9 because they required creative work. The matter was referred to the European Court of Justice whose role is to give an authoritative interpretation of EU law. It made the point that the “criterion for protection is not satisfied when the setting up of the database is dictated by technical considerations, rules or constraints which leave no room for creative freedom”. In expressing this opinion, the European Court of Justice appears to eliminate any non-creative arguments for protection of this and similar databases. This is relevant to nomenclatural or taxonomic databases in which the format is dictated by technical considerations, rules, and discipline-specific data standards and which require intellectual effort and skill, but not creative originality. That is, such databases are unlikely to meet the criteria for protection.

### Application of legal context to names and compilations

#### Principles

The following principles of copyright protection are relevant to species names or compilations of names. (1). Copyright can be applied to works that are original, individual, new creations with respect to the form of the presentation. Copyright gives the owner the right to reproduce, distribute, or display the work, to make derivative versions, to transfer those rights to others, or to license others to do some or all of the above. (2). Copyright does not cover ideas, procedures, systems or content. Copyright protection is appropriate only if the content is expressed in an original way. (3). Copyright law refers to ‘works’ - permanent or semi-permanent authored products that are in a form that can be perceived, reproduced, or communicated for more than a transitory period of time. Facts, ideas, opinions, and discoveries are not works and are not protected by copyright. (4). A non-copyrightable work or compilation remains non-copyrightable even if the author or others chose to mark it with a copyright (©) sign or with a Creative Commons license. (5). The initial owner of copyright is the creator of the work. In most European countries, only a person can be an author but a publisher or university can acquire rights by various legal instruments. Under U.K. database rights, if the database is made by an employee, and there is no agreement to the contrary, the employer is regarded as the maker. Similarly, U.S.A. copyright law indicates that if the creator has carried out the work for an employer with an agreement over ‘work for hire’, the rights are assigned to the employer. (6). If a work has no known author, the copyright protection is restricted to a period of 50 years after the publication. In such cases, the publisher is entitled to represent the unknown author (art. 7 (4) Berne Convention).

#### Taxon names are not copyrightable

Scientific names of species follow a standard binomial, Latinized format. These may be followed with the author and date of publication. Even when a name is new, the form of expression follows a well-established pattern. Taxon names are therefore not copyrightable. The collective of characters, spaces and punctuation that makes up a name of a taxon is regarded as a fact. The exclusion of names from copyright coverage is explicitly stated by the US government [47].

#### Taxonomic treatments are not copyrightable

Taxonomic treatments and descriptions of species are not copyrightable because they lack creativity of form. Rather, they are presented with a standardized form of expression for better comprehension.

#### Compilations

Title 17 of the US Code (U.S.C. 17) refers to compilations as works formed by the collection and assembling of preexisting materials or of data. Compilations are copyrightable only if they are original in their form of expression, for example with regard to the selection criteria, form of presentation, or system of classification. Compilations of names follow familiar formats to ensure that compilations are comparable with the products of other taxonomists. They are not original in the meaning of copyright law and therefore not copyrightable. Similarly, checklists and classifications that list species using widespread conventions cannot be subject to copyright restrictions.

#### Agreements

Irrespective of intellectual property rights, authors, publishers and holders of data may regulate the use of information through data use agreements. The agreements may be ambiguous if they are implicit. This is best eliminated if agreements are made separately and explicitly with each user.

#### Patents

Copyright is one a variety of intellectual property rights. Patents deal with rights that relate to inventions. Those who build names-based informatics tools should be aware that several patents have been applied to inventions associated with scientific names: Merging taxonomic information, Inventors Remsen, D. and Norton, C. US patent number 7,650,327 B2 (Jan 19, 2010) [48]; Systems and methods for resolving ambiguities between names and entities, US patent 7,925,444 B2, Inventors G. Garrity and C. Lyons (April 12, 2011) [49]; and Semiotic indexing of digital resources, Inventors Parker, C. T. and Garrity, G. M., US Patent application 20130013603 A1, (Jan 10, 2013) [50].

### The blue list: components of names and taxonomy that are not subject to copyright

‘The blue list’ is our attempt to identify those elements that may reasonably be expected to occur in checklists, classifications, taxonomies, and monographs. As familiar components, their inclusion lacks the creativity that makes copyright applicable. That is, the elements listed below may be freely re-used unless restricted by a use agreement. The list may not be complete.

● A hierarchical organization (= classification), in which, as examples, species are nested in genera, genera in families, families in orders, and so on.

● Alphabetical, chronological, phylogenetic, palaeontological, geographical, ecological, host-based, or feature-based (e.g. life-form) ordering of taxa.

● Scientific names of genera or other uninomial taxa, species epithets of species names, binomial combinations as species names, or names of infraspecific taxa; with or without the author of the name and the date when it was first introduced. An analysis and/or reasoning as to the nomenclatural and taxonomic status of the name is a familiar component of a treatment.

● Information about the etymology of the name; statements as to the correct, alternate or erroneous spellings; reference or citation to the literature where the name was introduced or changed.

● Rank, composition and/or apomorphy of taxon.

● For species and subordinate taxa that have been placed in different genera, the author (with or without date) of the basionym of the name or the author (with or without date) of the combination or replacement name.

● Lists of synonyms and/or chresonyms or concepts, including analyses and/or reasoning as to the status or validity of each.

● Citations of publications that include taxonomic and nomenclatural acts, including typifications.

● Reference to the type species of a genus or to other type taxa.

● References to type material, including current or previous location of type material, collection name or abbreviation thereof, specimen codes, and status of type.

● Data about materials examined.

● References to image(s) or other media with information about the taxon.

● Information on overall distribution and ecology, perhaps with a map.

● Known uses, common names, and conservation status (including Red List status recommendation).

● Description and/or circumscription of the taxon (features or traits together with the applicable values), diagnostic characters of taxon, possibly with the means (such as a key) by which the taxon can be distinguished from relatives.

● General information including but not limited to: taxonomic history, morphology and anatomy, reproductive biology, ecology and habitat, biogeography, conservation status, systematic position and phylogenetic relationships of and within the taxon, and references to relevant literature.

It would appear that no copyright law is infringed if a user extracts elements of the blue list from material that lacks legitimate user agreements. The list does not include images because the status of images that follow a familiar pattern is not clear. The elements of this list are rarely presented as unembellished flat lists. They often form part of web sites, of articles, of monographs, and so on. Even if the elements in the list are not copyrightable, the web site or monograph may be protected by copyright or database rights. If the processes by which content is extracted require the reproduction of copyright protected parts of the source, then the user has to respect those rights either by obtaining individual authorization or by using appropriate legal exceptions and limitations. Agosti and Egloff [51] provide a useful example of how non-copyrighted content may be extracted from copyright protected texts but remain compliant with the applicable copyright law.

### Perceptions of biologists and the law

Many compilers of taxonomic content claim that, or act as if, they hold intellectual property rights over taxonomic content. They use the argument of copyright law to impose conditions of use (Table 1). Rarely do the terms discriminate between creative design and facts, or distinguish copyright from database rights, or indicate which elements are covered by which licensing agreements. This suggests mis-conceptions that effort, intellectual opinion, or database construction merit copyright protection.

The consensus of opinions expressed through Taxacom [52] was that content should be freely and openly available. Some expressed frustration with restrictions on the use of data; with one contributor pointing to the US-based Scholarly Publishing and Academic Resources Coalition that supports the Budapest definition of Open Access: “By open access, we mean its free availability on the public internet, permitting any users to read, download, copy, distribute, print, search or link to the full text of these articles, crawl them for indexing, pass them as data to software or use them for any other lawful purpose…” [53]. Some submissions pointed to ITIS and GRIN [54,55] who impose no restrictions on data re-use.

In its submission to the workshop [56], Kew Gardens made the point that licensing is a means to satisfy a variety of needs. They include issues of credit for data providers, respecting the wishes of data providers in respect of data sharing, determining usage patterns, quality issues, building collaborations, and identifying commercial opportunities. The Society for the Management of Electronic Biodiversity Data (SMEBD) also emphasized that the assertion of rights is less about intellectual property rights, but more about giving due and appropriate credit for the efforts of content providers. The use of intellectual property or database rights as a mechanism to secure credit is not a legitimate use of those rights. It confuses the situation as to how a specific legal instrument should be used and creates uncertainty. Other options, such as agreements, are available.

### Attribution

The desire for credit over digital content aligns well with traditional practices in which information is extracted from printed scientific literature, evaluated, and combined with information from other scientific sources and with new, original research or opinions in order to assemble a new statement, and sources are explicitly identified. The paper-based tradition does not involve any copyright barriers, but expects that recognition will be given to any and all sources of information. Credit also assists users in assessing the reliability of content. How then might we ensure credit for re-use of digital content [57].

We identify three categories of credit: (1) scholarly citation of prior work; (2) legal obligations that arise from licensing agreements; and (3) ‘community credit’ that applauds those who are instrumental in collecting or aggregating data. The third process may involve no scholarly or critical input and so may conflict with the tradition of scholarly citation. Indeed, some submissions to the workshop suggested that aggregators receive disproportionate credit [52]. Despite this concern, any agent without whom data stop moving plays an important role. All contributors to the supply chain who seek credit should acquire credit. The concern for disproportionate credit can be addressed with a mechanism that gives authors greater credit than intermediaries.

Failure to give scholarly citation is plagiarism. Plagiarists may face considerable sanctions - papers may be withdrawn, university degrees retracted, or university staff dismissed [58-60]. Concern over the potential for plagiarism leads some players not to share content, but this harms data flow and impedes scientific progress.

At this time, taxonomists do not have effective mechanisms to track re-use of digital content. It is assumed that each actor should minimally credit the immediate sources on which their own work depends. The immediate source may not be the most important element of a supply chain, as names and other content may pass from original authors, to taxonomists who thoughtfully review particular clades, to those who non-critically compile taxonomic data for a broader taxonomic area, to the large aggregators which harvest this information in their efforts to build global compilations, to web sites that draw on taxonomic information from the aggregators, to subsequent users who mash up content from many sources, and so on. The domain is in need of a system of attribution that automatically can track use and reuse through diverse and long pathways of content flow.

Two technical strategies seem feasible to achieve this, annotation and nanopublication [61-64]. Both approaches involve assigning a universally unique identifier (UUID) to a content element. An attribution infrastructure might involve a browser plug-in that is downloaded with taxonomic content, reports the receipt and ongoing transfer of each element back to an annotation center, which keeps an automated tally of transactions and re-use. Each transaction adds a new actor to a provenance record that is linked to the identifier. As a subset of content moves from one place to another (is being re-used), any actors already in the provenance file are assigned an increment of credit. This process recognizes everyone in the supply chain but ensures that those near the origin, the authors, gain the greatest number of credits. An infrastructure involving devices such as this would allow the metrics to be accessed through the UUID and be made available in a citable form. If any piece of content is held by more than one originating author, then all authors should receive equal credit.

### Prospective

We presume that the infrastructure for the ‘Big New Biology’ will be modular, and that each module will include a core that takes responsibility for acquiring content from providers and will take responsibility for combining the information, adding value to it, and making it easily and simply available to end users in standard formats [65]. A node within a names-based cyberinfrastructure could take on responsibility for sharing names and taxonomic content, provide the service of capturing usage information, and return citable usage metrics to providers. The development and maintenance of infrastructure is a growing challenge for biodiversity scientists. The prevailing funding model that delivers short-lived research grants to individuals and small teams is unlikely to lead to the robust and reliable services that we expect of an infrastructure. A new paradigm is needed. The requirements of a system to manage names are now reasonably clear. This study has established that there are no copyright impediments to the sharing of names and related data. The system must reward those who make the contributions upon which we rely. Building an attribution system remains one of the more urgent challenges that we need to address together.

## Competing interests

The authors declare that they have no competing interests.

## Authors’ contributions

DJP: conceived of paper and wrote basic outline. WE: legal perspective and critical input. DA: critical input. DE: critical input. NF: critical input. GH: Critical input. JR: Critical input. DR: Critical input. All authors have read and approve the manuscript.
